# A Study on the Influence of the Chemical Nature of Fillers on Rheological and Fatigue Behavior of Bitumen Emulsion Mastic

**DOI:** 10.3390/ma13204627

**Published:** 2020-10-16

**Authors:** Ahmed Al-Mohammedawi, Konrad Mollenhauer

**Affiliations:** Engineering and Maintenance of Road Infrastructure, Faculty of Civil and Environmental Engineering Kassel, University of Kassel, 34127 Kassel, Germany; k.mollenhauer@uni-kassel.de

**Keywords:** active filler, bitumen emulsion mastic, dynamic shear rheometer, fatigue cracking resistance

## Abstract

Cold Bitumen Emulsion (CBE) mixture technologies have been recently developed to lower pavement construction temperatures to reduce environmental costs and control gas emissions. Due to its poor early mechanical strength, active fillers (i.e., cement) have been used to obtain high early stiffness in order to have the potential for timely construction of the next layer. There is, however, a lack of understanding about the impact of active fillers on the viscoelastic behavior and fatigue damage resistance of CBE mastics. This study, therefore, aims to identify the influence of active fillers on the rheological properties and the resulting fatigue behavior of CBE mastic, supported by chemical analysis for the filler-bitumen emulsion. For this aim, bitumen emulsion was mixed separately with seven fillers/blended fillers to prepare the CBE mastics. Various experiments, including continuous pH monitoring tests (chemical reactivity of filler-bitumen emulsion), Strain Sweep (SS) tests, Temperature-Frequency Sweep (TFS) tests, Time Sweep (TS) tests, and Linear Amplitude Sweep (LAS) tests were conducted on the CBE binder and the prepared mastics. Results show that the rheological performance and the fatigue damage resistance depend not only on the filler inclusions but also on filler type and chemistry. On this basis, the rise in complex shear modulus and the decrease in the viscous component is associated with a significant enhancement in fatigue performance for specific fillers.

## 1. Introduction

**Figure 1:**
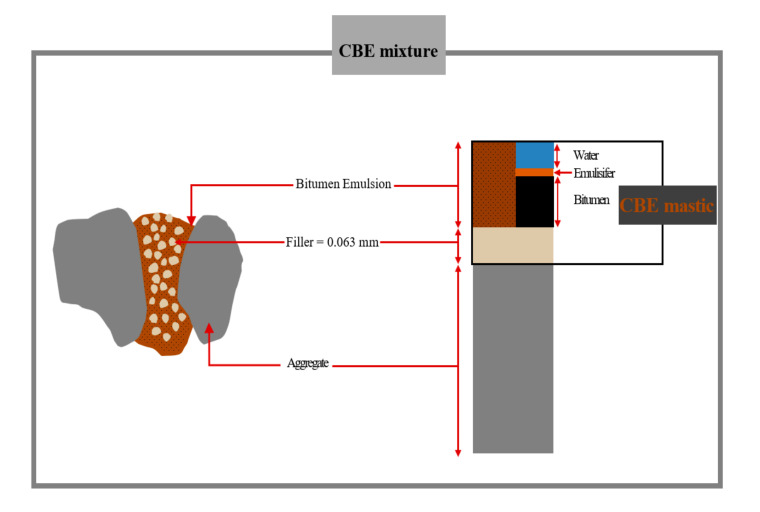
A schematic showing the Cold Bitumen Emulsion (CBE) mixture components.

The awareness towards sustainability and environmental issues in pavement construction has led to research focusing on developing green asphalt pavement technologies, ultimately aiming at a significant environmental, social, and economic benefit. One promising environmentally-friendly technology in pavement construction is the Cold Bitumen Emulsion (CBE) mixture. CBE mixture is constructed at ambient temperature using virgin or reclaimed asphalt pavement (RAP) aggregate (or a combination of both) mixed with a bitumen emulsion. The coarse and fine particles are bonded by a thin film mastic (illustrated in Figure 1) consisting of Bitumen Emulsion (BE) and mineral fillers. Therefore, CBE technology contributes to reducing energy consumption, saving natural resources, and preserving the environment [1,2].

The CBE mixture’s strength development and rheological properties are highly linked to its mastic characteristics, as different physiochemical processes (i.e., curing processes) occur within the mastic matrix, which affect the whole mixture properties in both fresh and in-service states. In this context, the mastic can be modified by various additives, such as active fillers. The addition of mineral fillers can significantly change the rheological response and fatigue damage resistance of the mastic [3,4,5]. In general, mineral fillers can be chemically grouped into active and inactive (or inert) fillers, depending on their reactivity within bitumen emulsion. Inert fillers, such as limestone, are classified as inactive fillers because of their mineral composition and are usually employed as a stiffness regulator by adding solid particles to the bitumen matrix. As little or no chemical reaction is expected, it maintains the viscoelastic response [6]. An active filler is defined as a filler that reacts with water or with the bitumen itself (altering bitumen structure) [7]. The main chemical compounds in the active fillers are calcium oxide (CaO) and silicon dioxide (SiO_2_), which are responsible for forming the co-binder (hydraulic binder) within the CBE mixture. During the hydration process, the CaO compound reacts with water and provides the mastic with pozzolanic properties, which are responsible for accelerating the curing process due to the formation of Ca(OH)_2_ [8]. Besides, filler with SiO_2_ compound has a tendency to make dense microstructures within the CBE mixture, leading to lower air voids and, therefore, higher strength, but delaying the curing process [9]. The coexistence of CaO and SiO_2_ together in filler leads to the formation of the calcium silicate hydrate (CSH) phase in the form of geopolymer gel, which has been proven to enhance the strength development of the final product [10]. Furthermore, filler with both earlier mentioned compounds and Al_2_O_3_ compound can form the CASH phase, that improves strength with short setting time [11]. On the other hand, the Fe_2_O_3_ compound has been found to have an undesirable impact on the compressive strength [12]. Accordingly, fillers should be designed to obtain the required mechanical and rheological characteristics, considering the mixture type (HMA or CBE).

CBE mastic behavior and structure are different from that of HMA, as CBE comprises water in its system to have reduced viscosity for mixing and compaction. In CBE mixtures, the water needs to be evaporated to enable durable bitumen aggregate adhesion and high bearing capacity. Active fillers can be added to CBE mixtures to accelerate the curing process. Consequently, the water and active filler react and produce hydraulic binder, forming rigid structures within the CBE mixtures. In this way, fillers improve the early mechanical properties but can lead to rigid long-term properties. Generally, fillers strengthen bitumen through the three main mechanisms [13,14,15] of particle geometry, volume filling, and chemical interaction. Particle geometry and volume filling are considered as mechanical reinforcement [15,16]. Volume filling is caused by adding more solid particles to the system, resulting in high stiffness, and chemical interaction includes the chemical composition and alkalinity of fillers.

Nevertheless, the response of modified CBE mastic with active filler may move from the viscoelastic range to pure elastic. Thus, CBE mastic could show low fatigue and fracture resistance under high load cycles [17]. Therefore, the mechanism of filler reinforcement in CBE mastic plays a major role in controlling the distress and failure mechanisms in the CBE mixture [18]. During its service life, the CBE mixture is subjected to different distress. One of the most critical failures threatening CBE mixture can be fatigue cracking. Firstly, this failure appears as microdamage in the mastic and then, with increasing load cycles, grows up into macro cracks [19]. Thus, the physiochemical and rheological characteristics of CBE mastic control the stress–strain distribution mechanism under thermal and traffic loading. In this research area, Anderson and Goetz [20] conducted a study on different fillers in HMA mastic, concluding that different fillers had different reinforcing effects, depending on the nature of filler. However, in an extensive study on dust collector fines, Anderson et al. [21] came up with a conclusion that the nature and extent of the physiochemical interaction needed further study. Other researchers have tried different methods to study the physiochemical interaction in terms of the effect of the filler chemical composition on HMA mastics and/or asphaltic mixtures. Kandhal [22] measured the pH values of a diluted water solution of fillers and stated that the pH values could hardly be related to how filler changes the HMA mastic behavior. Recently, Ziyani et al. [23] chemically investigated the reactivity of fine aggregate in bitumen emulsion using the rise in pH test and element dissolution.

However, the physicochemical interaction between active fillers and bitumen emulsion and its effect on rheological performance and fatigue damage resistance has yet to be well investigated. In difference to HMA mastics, the availability of water in CBE mastics during curing process enables the development of mineral bonds and structures within the mastics.

For bitumen emulsion, the physicochemical interaction starts at adding the destabilizing agent (filler) to the bitumen emulsion medium, initiating the breaking, and, therefore, accelerating the coalescence of bitumen droplets due to the increase in salinity (pH increase). The increase in salinity occurs due to a decrease in the concentration of free H+ in the solution because of ionic exchange between H+ and alkali species from minerals such as Ca++ (and also between the emulsifier molecules and the carbonate anion). The result of this reaction is a specific form of salt that can be absorbed by the interface region and improve adhesion properties [24,25]. This mechanism affects the bitumen-filler affinity in terms of controlling the contact angle, surface tension, adhesion work, and cohesion between bitumen and fillers, which could affect the crack development mechanism (adhesive failure at the filler-bitumen interface region or cohesive failure within the bitumen) [26]. In this context, incorporating fillers could either manage the crack failures within the mastic matrix or, in some cases, make CBE mastic very stiff and sensitive to fatigue cracking [27,28].

To characterize the fatigue damage resistance of mastic, various testing methods and approaches have been explored within the time fatigue sweep tests, such as 50% reduction in the complex shear modulus [29,30], the Drop In Phase Angle (DPA) [31], the Dissipated Energy Ratio (DER), the Ratio of Dissipated Energy Change (RDEC) [31,32], and Energy Stiffness Ratio (ESR) [33,34]. Using a different concept, Wen and Bahia [35] developed a binder fatigue testing procedure based on accelerated damage development in the specimen by means of applying a rapidly increased loading amplitude (strain). The raw data was interpreted by Viscoelastic Continuum Damage (VECD) mechanics theory principles, and then the fatigue law was obtained. A critical review of previous studies shows that fatigue resistance studies are somewhat restricted to CBE mixtures, knowing that the mastic is the actual binder in the CBE mixtures. This research provides a comparative investigation on the chemical reactivity of active fillers and its consequences on the rheological properties and fatigue damage resistance of CBE mastics, employing Strain Sweep (SS) tests, Frequency–Temperature Sweep (FTS) tests, strain-controlled Time Sweep (TS) tests, and Linear Amplitude Sweep (LAS) by Dynamic Shear Rheometer (DSR). Taking into consideration that each filler could influence the mastic fatigue-based properties in different fatigue-induced damage patterns.

## 2. Materials and Methods

### 2.1. Basic Materials

The mastic specimens were prepared by mixing cationic bitumen emulsion type C60B10-BEM of penetration grade 70/100 with seven fillers/blended fillers. A relatively low mass ratio of 0.21 (filler to residual bitumen) was chosen to avoid the particle to particle contact phenomenon (diluted medium). Thus, the filler particles and the effective bitumen film around the filler particle were dispersed and not expected to contact each other. Seven fillers were used and mixed with BE, namely: limestone (LS), cement (CE), ladle slag (LD), silica fume (SF), ettringite (ET), geopolymer with an activator (GE), and geopolymer without activator (GO). ET filler is a combination of 70% LD and 30% Gypsum, as suggested by Nguyen et al. [36]. GO filler includes 55% LD, 35% Fly ash, and 10% SF. GE filler was prepared by mixing GO filler with a 3.5% activator by its weight. The activator was a combination of 50% sodium hydroxide (NaOH) in the concentration of 10 M and 50% sodium silicate as an alkaline activator. All used filler particles were smaller than 63 μm. The Ring and Ball (R&B) test was performed to determine the softening point temperatures of the basic bitumen and mastics. Thereafter, the difference between the R&B temperatures (∆ R&B °C) of the basic bitumen and each mastic was determined. The density and chemical composition of the fillers and the ∆ R&B °C are given in Table 1.

### 2.2. Mastic Preparation

**Figure 2:**
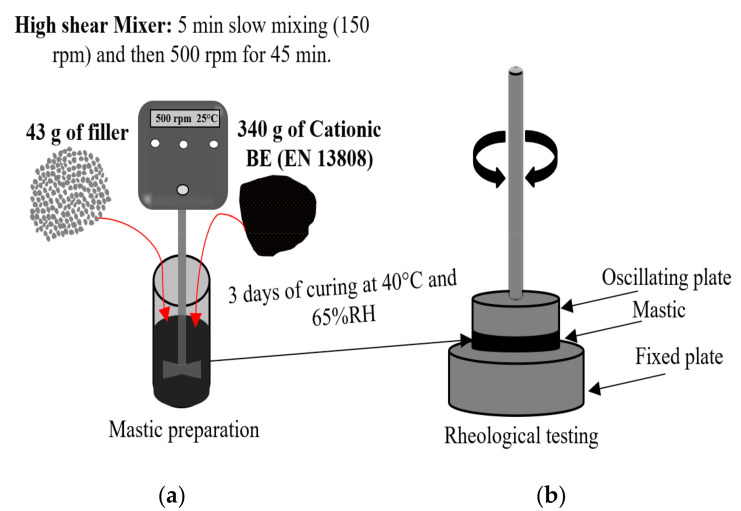
A schematic showing: (**a**) Mastic mixing and preparation; (**b**) Dynamic Shear Rheometer (DSR) rheological testing.

In this paper, 43 g of filler was incorporated and mixed gradually with 340 g of CBE binder at ambient temperature, employing a mechanical mixer. The mixing regime, as shown in Figure 2a, was 5 min slow mixing with a speed of 150 rpm to initially agitate the filler particles into the CBE medium, and then a mixing speed of 500 rpm was carried out for 45 min. This mixing process was also carried out to the CBE binder to avoid possible effects on results. During mixing, no particle separation was noticed. However, at the end of the mixing process for specific fillers, breaking was observed. The resulting mixture was then poured into a shallow glass dish to avoid bubbles due to water evaporation. After 3 days of curing with 40 °C and 65% relative humidity, small specimens (approximately 8 mm diameter) were cut for DSR testing, as shown in Figure 2b. DSR parallel plates were heated to 64 °C, and then the mastic specimens were positioned on the lower plate. The upper plate, therefore, was lowered down to reach 2 mm thickness. After that, the unnecessary edges were trimmed.

### 2.3. Experimental Program

Various tests were performed using DSR, with an 8 mm parallel plate geometry and a 2 mm gap, including SS, FTS, TS, and LAS tests. Furthermore, continuous pH monitoring tests were performed using a pH meter. Each test is described in the following sections:

#### 2.3.1. Strain Sweep (SS) Test

SS tests were initially performed to determine the Linear Viscoelastic (LVE) limit and define a suitable range of deformation levels. SS tests were conducted at −20 °C, with a constant frequency of 10 Hz. From the results, a strain of 0.05% was found appropriate as per the LVE limit.

#### 2.3.2. Frequency–Temperature Sweep (FTS) Test

FTS tests were used to assess the CBE binder and mastics’ rheological characteristics using a constant strain amplitude of 0.05%, with frequencies ranging from 0.1 to 10 Hz and temperatures from −20 to 40 °C. The raw data was interpreted in the form of master curves [37,38]. The master curves were produced using the sigmoidal model following Equation (1) along with shift factor using the Time–Temperature Superposition Principle (TTSP), following Arrhenius law Equations (2) and (3).

(1) $$G*=G_{0}^{*}+\frac{G_{\infty }^{*}}{1+e^{-\left(\frac{ln\;fr-f_{0}}{z}\right)}}$$

(2) $$\alpha_{T}=e^{\frac{-\Delta H}{R}\left(\frac{1}{T}-\frac{1}{T_{ref}}\right)},$$

(3) $$fr=\alpha_{T}*fi,$$

where $G^{*}$, *f_r_*, $G_{0}^{*}$, *F*_0_, $G_{\infty }^{*}$, z, $\alpha_{T}$, *R*, Δ*H*, $T_{ref}$, and $f_{i}$ are the modeled complex shear modulus, the reduced frequency, the minimum limiting complex shear modulus, the min limiting frequency, the span of complex shear modulus value, the fitting parameter, the shift factor, the universal gas constant, the activation enthalpy for flow, the reference temperature (20 °C), and the testing frequency, respectively.

#### 2.3.3. Time Sweep (TS) Test

**Figure 3:**
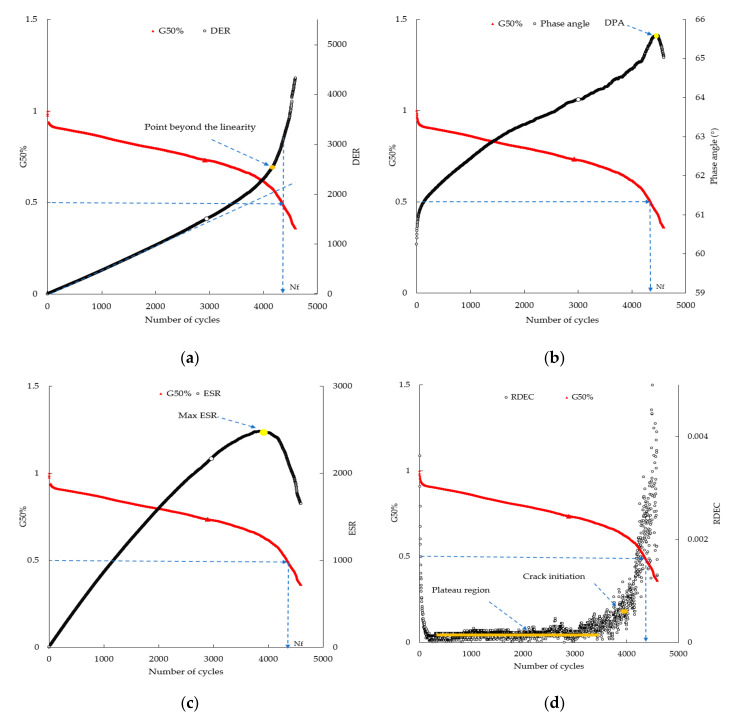
Comparing Nf for various approaches with the G50%: (**a**) Drop In Phase Angle (DPA); (**b**) Dissipated Energy Ratio (DER); (**c**) Ratio of Dissipated Energy Change (RDEC); (**d**) Energy Stiffness Ratio (ESR).

Strain-controlled TS tests were carried out to assess the fatigue resistance of CBE mastics. The tests were conducted at 20 °C temperature and 10 Hz of loading frequency with three different unique strain levels, 1.5%, 2.5%, and 3.5%, which made fatigue failure occur in a reasonable time. Before the test, the testing duration was undetermined, and thus, in some cases, binders could take several hours to reach adequate damage to provide a clear impression regarding their fatigue performance. Time sweep data for the tested CBE mastics were analyzed by five different approaches detailed below. The first approach is traditional fatigue definition. This approach is defined as the number of cycles to reach the 50% reduction in complex shear modulus (G*_50%_). Figure 3 illustrates the number of cycles of a load to cause a 50% reduction in complex shear modulus versus other approaches. The second approach is the Drop In Phase Angle (DPA). DPA is commonly used in the literature to identify fatigue life. DPA and G*_50%_ are plotted and shown in Figure 3a. It can be noticed that there is a sensible drop in the phase angle after a steady increase. The drop in phase angle indicates the change in the proportion of complex modulus reduction.

The third approach is Dissipated Energy Ratio (DER). In the time sweep test, the Dissipated Energy (DE) per cycle can be obtained using Equation (4), and therefore, the DER is calculated using Equation (5).

(4) $$DE_{i}=\pi \varepsilon^{2}G_{i}sin\delta_{i}$$

(5) $$DER=\frac{\left(\sum_{i=1}^{N}DE_{i}0\right)}{DE_{n}}$$

where $DE_{i}$, ε, $G_{i}$, $\delta_{i}$, $\sum_{i=1}^{N}DE_{i}$, and $DE_{n}$ are the dissipated energy at cycle i, the strain level, the complex modulus at cycle i, the phase angle at cycle i, the accumulated dissipated energy up to cycle n, and the dissipated energy at cycle n, respectively. Figure 3b shows the DER plotted against the number of load cycles and compared with the G_50%_ definition. The number of cycles to cause failure is the point on the curve at which the curve becomes nonlinear. Increasing load cycles results in a steeper slope in the curve; thus, all the properties change very rapidly. The fourth approach is the Ratio of Dissipated Energy Change (RDEC). The RDEC approach is applied to eliminate the bias effects of dissipated energy (i.e., heat generation) and can be calculated using Equation (7). It has been suggested that RDEC is a good indicator to define fatigue failure in bituminous materials [32].

(6) $$RDEC=\frac{\left(DE_{i}-DE_{i+1}\right)}{DE_{i}},$$

where *RDEC*, $DE_{i}$, and $DE_{i+1}$ are the ratio of the dissipated energy change per load cycle, the dissipated energy at the cycle *i*, and the dissipated energy cycle *i* + 1, respectively. In Figure 3c, RDEC values are plotted against the number of cycles and compared with G_50%_ criteria. A plateau trend line can be noticed from the plot, indicating a constant quantity of energy turned into damage in the materials. The average RDEC values in the plateau region are defined as the plateau value (PV). The higher the PV, the higher the energy amount being transformed to damage and, consequently, the lower the fatigue life. The crack initiation can be identified when the RDEC values increase after the PV. This means that the material is damaged and is no longer able to hold more cracks. The last approach is the Energy Stiffness Ratio (ESR). ESR is a relatively new definition for fatigue life failure that has been developed by Mitchell et al. [33] based on work done by Rowe and Bouldin [39]. ESR can be calculated as follows:

(7) $$ESR=\left(\frac{G_{i}^{*}}{G_{0}^{*}}\right)*N_{i},$$

where *ESR*, $N_{i}$, $G_{i}^{*}$, and $G_{0}^{*}$ are the energy stiffness ratio, the number of cycles at *i* cycle, the complex modulus at the cycle *i*, and the initial complex modulus, respectively. Figure 3d shows the ESR values plotted against the number of cycles and compared with *G*_50%_ criteria. The peak in the ESR curve indicates the number of cycles to cause fatigue failure. From the resulting plots of the failure criteria versus strain level, fatigue lives are evaluated, and the fatigue is modeled by Equation (8).

(8) $$X_{i}=A\cdot \varepsilon_{i}^{B},$$

where *X_i_* is the failure parameter, *A* and *B* are the fatigue coefficients, and $\varepsilon_{i}$ is the applied strain.

#### 2.3.4. Linear Amplitude Sweep (LAS) Test

LAS test is an accelerated fatigue test introduced recently [40], in which the strain level is amplified systematically. The test starts by identifying the specimens’ undamaged linear viscoelastic properties by conducting Frequency Sweep (FS) testing. 

The FS test was performed utilizing 25 unique frequencies in the range of 0.1 to 30 Hz under 0.05% strain level. A relatively low strain level (0.05%) was used in this paper to ensure all mastics were with the LVE limit. After that, the specimens were tested with the Amplitude Sweep (AS) test straight after the FS test as no or little damage was expected during FS [41]. The specimen was tested within a cyclic load in the AS, and the strain level was amplified linearly from 0.1% to 30% under a 10 Hz frequency. LAS test was introduced as an accelerated fatigue test to reduce the testing time compared to the time sweep test [42]. The test results were analyzed using the VECD procedure to estimate the fatigue damage resistance. Schapery’s work potential theory (WPT) was used in the VECD approach to model fatigue damage growth [43]. Based on this theory, for viscoelastic material (bituminous material), work can be correlated to damage by:

(9) $$\frac{dD}{dt}={\left(-\frac{\partial W}{\partial D}\right)}^{\alpha },$$

(10) $$\alpha =\frac{1}{1+m},$$

where *W*, *D*, *α*, and *m* are, respectively, the work performed, the damage intensity, a material constant (the rate at which damage progresses), and the slope of a log-log plot of storage modulus (*G*’) against angular frequency (ω) that is derived from the first part of the test (frequency sweep test).

Based on Johnson (2010), the integrity parameter (C) and the accumulated damage (*D*) in the material due to the amplitude sweep (AS) loading can be predicted using Equations (11) and (12), respectively [41]:

(11) $$C\left(t\right)=\frac{G^{*}sin\delta }{G_{i}^{*}sin\delta_{i}},$$

where *C*(*t*), G* and *δ*, $G_{i}^{*}$, and $\delta_{i}$ and *t* are the integrity parameter, the complex shear modulus and phase angle at time *t*, the initial value of G* and *δ*, and testing time in seconds, respectively.

(12) $$D\left(t\right)=\sum_{i=1}^{N}{\left[\pi *\gamma 0^{2}(C_{i-1}-C_{i})\right]}^{\frac{\alpha }{1+\alpha }}{\left(t_{i}-t_{i-1}\right)}^{\frac{\alpha }{1+\alpha }},$$

where *D*(*t*), *γ0*, and *t* are the damage accumulation, the strain that is applied at a given data point (%), and the test time (s), respectively. The *C*(*t*)−*D*(*t*) relationship is established by the curve fitting technique using the power-law expression:

(13) $$C\left(t\right)=C_{0}-C_{1}{\left[D\left(t\right)\right]}^{C_{2}},$$

where *C*_1_ and *C*_2_ are coefficients of fitting curve equations, whereas *C*_0_ is the initial value of *C*(*t*). The fatigue law parameters *A* and *B* can then be determined using the following expressions:

(14) $$A=\frac{f{\left(D_{f}\right)}^{1+\left(1-C_{2}\right)\alpha }}{\left[1+\left(1-C_{2}\right)\alpha \right]{\left(\pi C_{1}C_{2}\right)}^{\alpha }},$$

(15) B=−2α

where *f* and $D_{f}$ are the loading frequency (Hz) and the damage accumulation at failure, respectively. $D_{f}$ can be defined as the damage accumulation (*D*(*t*)) value that makes a reduction of 35% in the integrity parameter (*C(t)*). In this study, 50% was used for comparison purposes. *D_f_* can be calculated using:

(16) $$D_{f}={\left({\frac{0.5}{C}}_{1}\right)}^{\frac{1}{C_{2}}},$$

#### 2.3.5. Chemical Reactivity of Filler-Bitumen Emulsion

**Figure 4:**
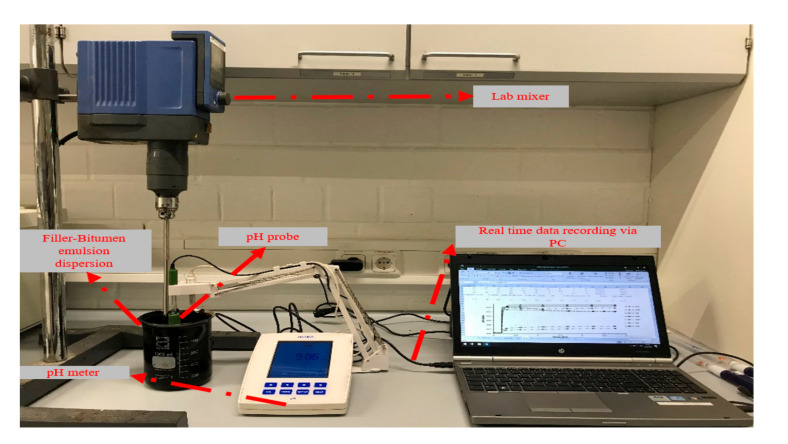
Experimental setup for continuous pH monitoring test.

The continuous pH monitoring tests were performed using a HANNA HI5221 pH meter connected with a PC for continuous monitoring, as shown in Figure 4. The testing procedure consisted of adding 100 g of filler to 600 g of bitumen emulsion at 25 °C temperature with frequent stirring at a speed of 150 rpm. The monitoring of the pH value over time started for the bitumen emulsion for 10 min and then continued to 2.7 h with filler addition. The real-time measurements were recorded, and the seven different fillers were tested separately.

## 3. Results and Discussion

### 3.1. Chemical Reactivity Analysis of Filler-Bitumen Emulsion

**Figure 5:**
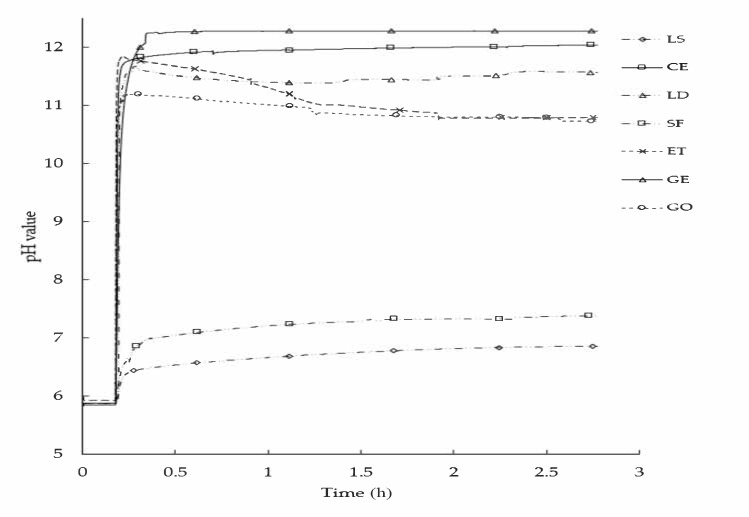
The chemical reactivity of filler-bitumen emulsion.

To provide a clear overview of the alkaline effect and chemical composition of fillers on bitumen emulsion, the chemical reactivity of filler-bitumen emulsion was continuously monitored in terms of the pH evolution and plotted in Figure 5. Generally, all used fillers increase the pH of bitumen emulsion as a function of time. However, each filler has a different influence on bitumen emulsion, depending on its chemical composition. Thus, the SF and LS fillers raise the pH smoothly and slightly from 5.8 (bitumen emulsion pH level) to 7 and 6.5, respectively, with a slight increase with time. This is due to their low ability to bind free H+ ions as both have low CaO content, and SF has high SiO_2_ content. In contrast, due to their high CaO content and low SiO_2_ content, GE, CE, LD, ET, and GO increase the pH dramatically to 12, 11.7, 11, 11.5, and 10.5, respectively. However, CE and GE fillers show a sudden increase in the pH value for the first few minutes until a constant pH value is reached. CE filler bonds a large amount of the free H+ ions in the bitumen emulsion, forming CSH and CASH phase materials. These phases are known as stiff hydration products, which offer extra strength to the mastics (detailed discussion provided in the next sections). While the use of activator in GE filler increases the pH value to 12 because the activator solution’s pH is higher than that of the material itself; therefore, it activates the filler material and then makes it able to bind more H+ ions. This observation can be clearly detected during the first few minutes when the pH increases smoothly as the filler and activator are added to the bitumen emulsion. However, ET filler shows a completely different trend, displaying a sharp increase in pH at the beginning because CaO first reacts with water, and then it exhibits a gradual decrease until reaching a steady-state, which is about pH 11. This trend is caused by the presence of gypsum (neutral pH value) as it counterbalances the pH level of the solution, forming an ettringite (ET) binder.

**Figure 6:**
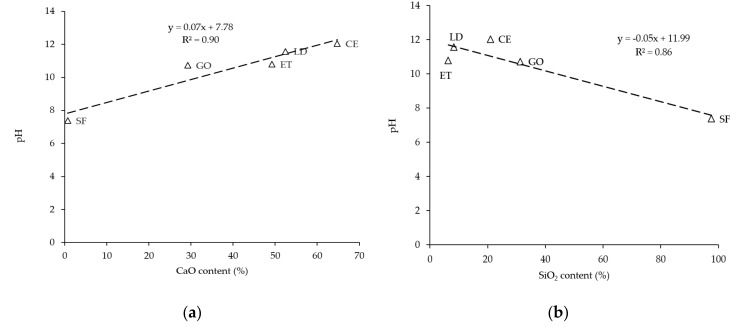
Correlation of pH of bitumen emulsion with: (**a**) CaO content %; (**b**) SiO_2_ content %.

Based on filler reactivity, a chemical analysis was established comparing the fillers’ chemical composition with their pH value in bitumen emulsion. In this regard, only the silicon dioxide (SiO_2_) and calcium oxide (CaO) were considered related to the analysis. The other chemical compounds were not considered in this analysis. In addition, LS and GE fillers were not taken into account because LS is an inert filler and does not have both earlier mention compounds, while GE has the same GO chemical composition, and pH is affected by the pH level of the activator. From Table 1 and Figure 5, linear correlations between pH change and CaO, and between pH change and SiO_2_, were established and plotted in Figure 6a,b, respectively. Figure 6a shows that the pH value increases as a function of CaO oxide. In this sense, the CaO content is noticeably higher for CE than other fillers, followed by LD, ET, GE, GO, and SF fillers. As expected, the SF filler has the lowest pH value as it has low CaO content and high SiO_2_ content. In contrast, Figure 6b shows a different trend as the pH value decreases as SiO_2_ increases. These observations illustrate that there is a strong, linear correlation between pH and filler chemical compound contents (CaO and SiO_2_). This remark explains why a particular filler has a specific pH level, which, in turn, has an effect on mastic rheological and fatigue behavior.

### 3.2. Mastic Rheological Performance

#### Master Curves

**Figure 7:**
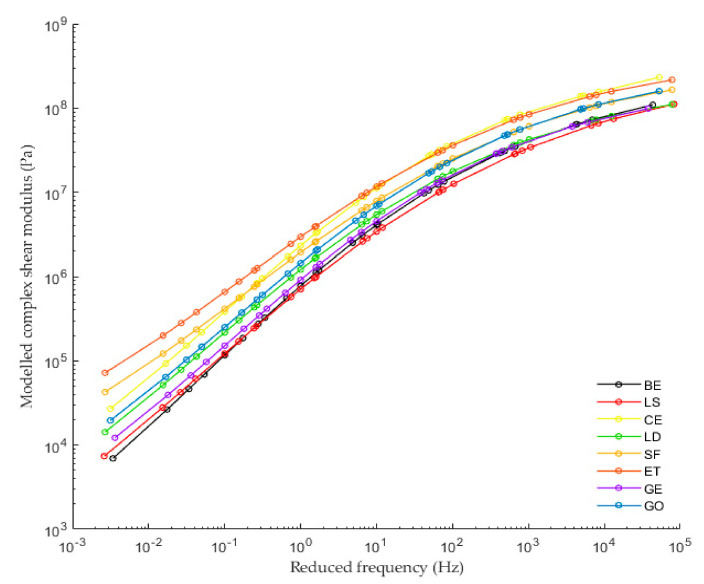
Modeled complex master curves of Bitumen Emulsion (BE) and CBE mastic at a reference temperature of 20 °C.

**Figure 8:**
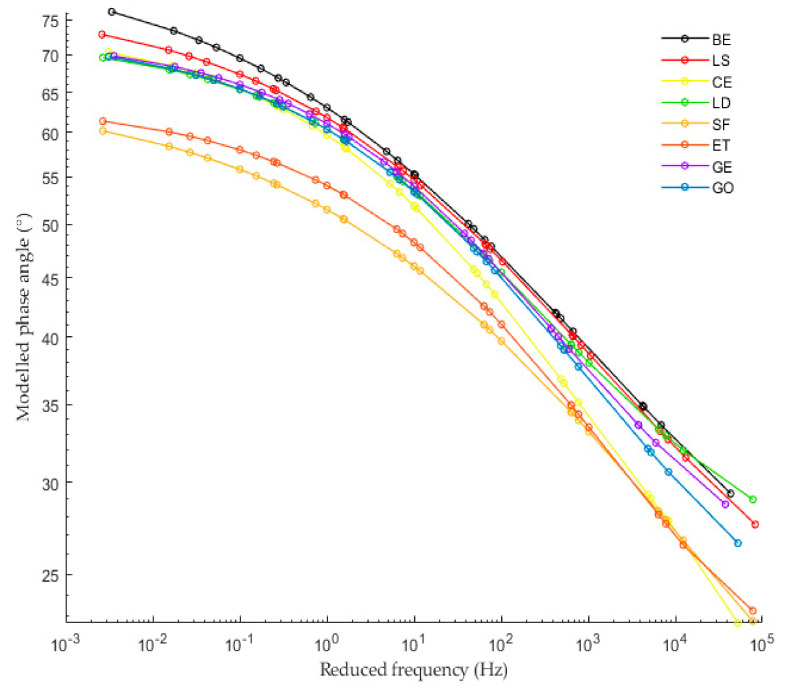
Modeled phase angle master curves of Bitumen Emulsion (BE) and CBE mastic at a reference temperature of 20 °C.

The complex shear modulus master curves were produced by utilizing the shift factor equation based on the TTSP and modeled by the sigmoidal. From Figure 7 and Figure 8, it can be observed that LS filler almost does not change the complex shear modulus and the phase angle of CBE binder at the whole range of frequencies. This observation could be due to the addition of an inert solid particle to the system with low concentration, thus maintaining the viscoelastic property.

Moreover, it is worth noting that the LD and GE fillers have a comparable influence on the CBE binder; both increase the complex modulus and decrease the phase angle at the whole range of frequencies in comparison with the CBE binder because both have the chemical compound that forms rigid phases (CSH and CASH). Mastic with GO filler exhibit higher complex shear modulus and lower phase angle than LD and GE at the whole range of frequencies. As expected, CE mastic exhibits higher complex shear modulus and lower phase angle than those of GE, LD, and GO, especially at high frequencies. Although ET and SF have different chemical compositions, they offer a relatively similar effect on CBE binder (SF has high density and ET has a crystallin network). They exhibit the highest stiffening potential and the lowest phase angle among all tested mastics over the whole frequency range. This remark, however, does not include CE in the low-temperature zone (high frequencies) as CE mastic has the highest complex modulus in that zone. This inversion conveys that CE mastic is stiffer than ET and SF. In this context, greater complex modulus and lower phase angle are not usually desirable at low temperatures because it makes the CBE mixture very sensitive to crack initiation and growth. A detailed discussion about crack damage resistance is described in the fatigue performance section.

### 3.3. Fatigue Performance

#### 3.3.1. Time Sweep Test

**Figure 9:**
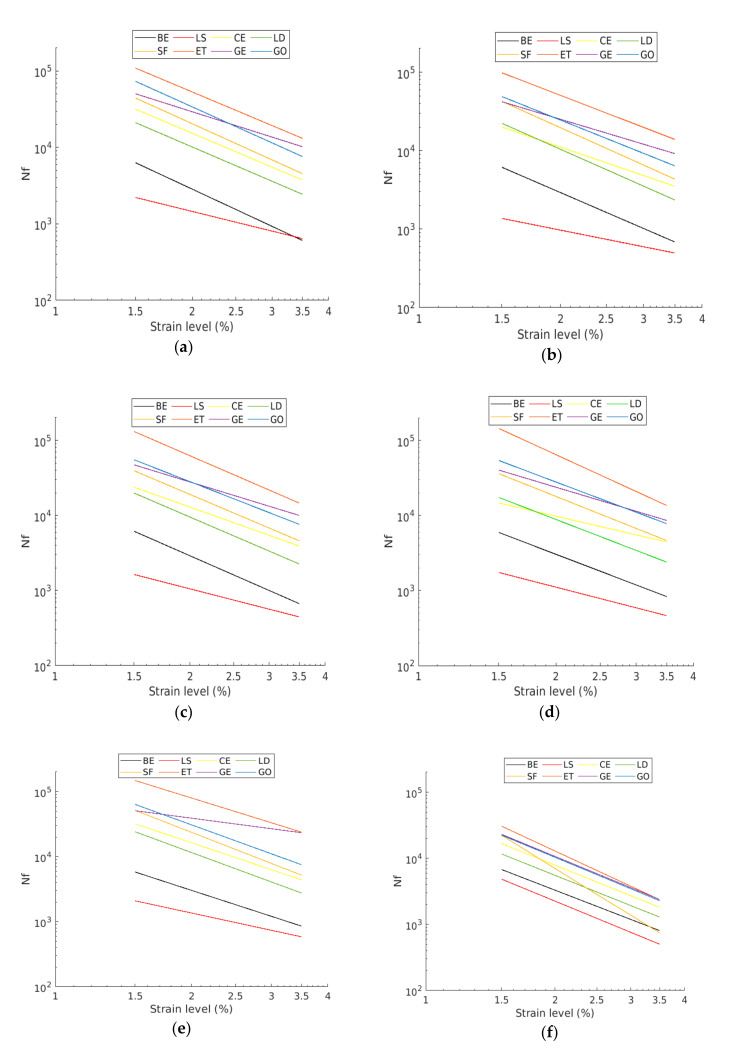
Fatigue laws derived from different failure approach: (**a**) G_50%_; (**b**) DPA; (**c**) DER; (**d**) RDEC; (**e**) ESR; (**f**) LAS.

In this paper, all CBE mastics were tested with a controlled strain time sweep test at a frequency of 10 Hz and under 20 °C temperature. According to the previously mentioned fatigue definitions, all tested mastics’ fatigue laws and lives were evaluated from the fatigue approaches and are summarized in Figure 9 and Table 2, respectively. There are slight differences in predicting fatigue lives from different fatigue approaches as each approach employs a different concept to define fatigue failure, as shown in Figure 2. According to the achieved results, it can be observed that the different mastics have different fatigue coefficients and, therefore, reach different numbers of load application to show failure. All mastics (except LS mastic) have fatigue resistance greater than that of the CBE binder. LS and ET corresponding mastics have the lowest and the highest fatigue life, respectively, compared with the rest of mastics. Therefore, the fatigue life of tested mastics can be ranked as follows: ET > GO and GE > SF > CE > BE > LS. 

#### 3.3.2. LAS Results Analysis

**Figure 10:**
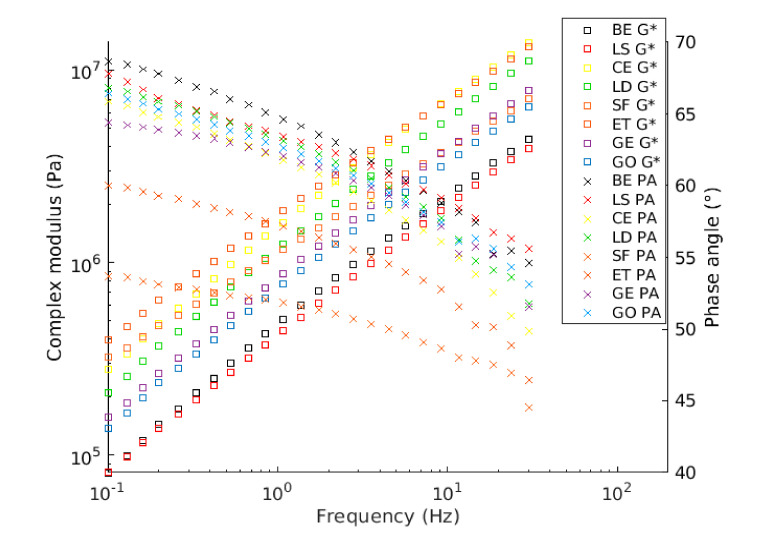
Frequency sweep output of Linear Amplitude Sweep (LAS) test at 20 °C.

The complex shear modulus (G*) and the phase angle (PA) over the tested frequency range resulting from the frequency sweep test of the LAS testing procedure at the testing temperature of 20 °C are graphed in Figure 10. The complex shear modulus increases as a function of frequency. It can be observed that the CBE binder has the lowest complex shear modulus when compared with the mastics. However, LS mastic has a complex shear modulus value, nearly similar to that of BE. It is interesting to notice that at lower frequencies, the viscous part of the CBE binder is higher than that of mastics; consequently, it has more time to relax than at a higher frequency region. This is due to the fact that the filler incorporation increases the elastic part and decreases the viscous part. Nevertheless, due to the shortage of time, the difference is less noticeable as the frequencies increase. In general, the results of FS are quite similar to the master curve results in Figure 7 and Figure 8.

**Figure 11:**
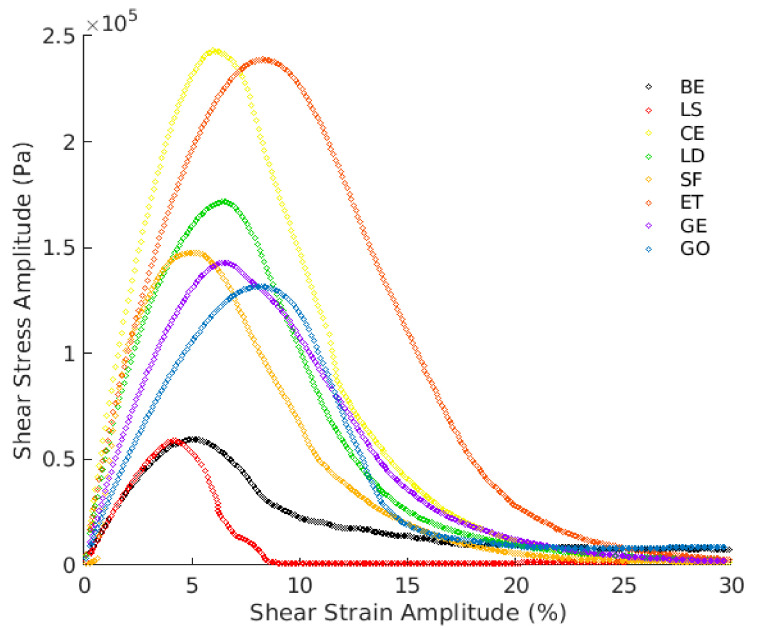
Shear stress–strain of different mastics.

**Figure 12:**
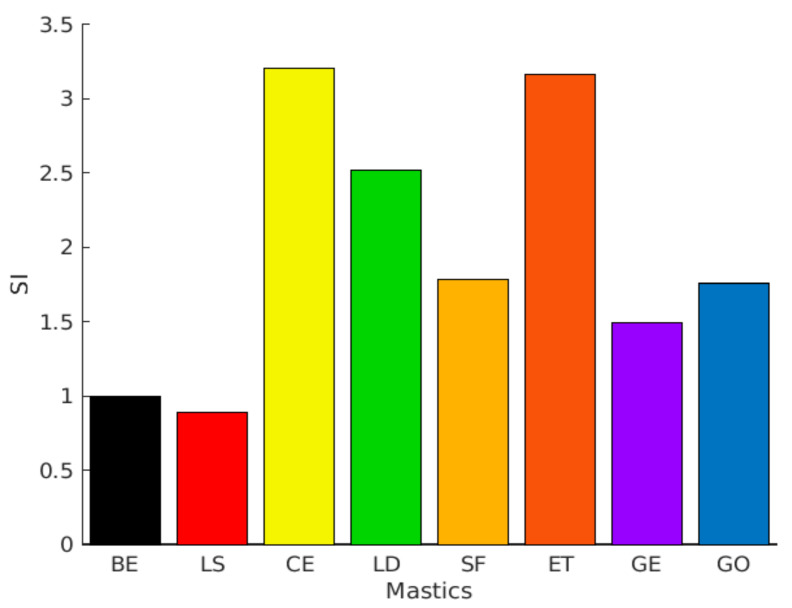
Stiffness Index (SI) of different mastics at 10 Hz under 20 °C.

Figure 11 shows the stress–strain relationship for the tested specimens. Three regions are characterized and used to identify the fatigue failure from the stress–strain curve: the strain value at the peak, the plateau value of the peak, and the steepness of the curve (before and after the peak). In the initial part of the curve, the shear stress increases linearly as the strain increases. An additional increase in the strain amplitude decelerates the growth of the shear stress. This is the point at which the specimens start exhibiting nonlinear behavior. Mastics reach the peak of the shear stress at various strain levels. After the peak zone, the shear stress drops down as the strain amplitude increases, implying substantial damage has been produced in the specimen. Thus, a sharp decrease in this region means fast induced damage. It can be seen that the peak stresses of the mastics are higher than the CBE binder. This behavior is related to the mastics’ higher complex shear modulus at 20 °C temperature, as previously depicted by the complex shear modulus and phase angle in Figure 10. Moreover, the calculated Stiffness Index ($SI=\frac{G_{mastic}^{*}}{G_{bitumen}^{*}}$) of each mastic is illustrated in Figure 12. Figure 11 and Figure 12 show that when SI decreases, the peak shear stress decreases because of the complex shear modulus reduction, which needs less stress to deform the material. However, not all mastics with high SI reach the peak of stress at a lower strain level (such as CE mastic). This indicates that carrying capacity before failure depends on the filler type and interaction with the bitumen. Normally, greater complex shear modulus creates an increase in the stress level, and, therefore, the specimen bears a smaller amount of damage at failure (behaves as fragile material). This effect is, nevertheless, a filler type concern. According to the above, mastic with LS as inactive filler shows a sudden drop in shear stress after a relatively small plateau at 4.5% strain value, which is not seen for the rest of mastics; whereas, without filler (CBE binder), the curve continues with smooth falling at 5% strain. As the LAS test is an accelerated test, the used strain amplitudes exceed the linear viscoelastic region of mastics (and bitumen), and the strain dependence changes with the filler type. Therefore, the post-peak part of specific mastics shows discrepancies (nonlinear behavior).

This can be clearly detected with LS mastic (and also for CE mastic). With SF filler, the mastic exhibits a sudden increase with a short peak, and then starts decreasing at 5% strain, indicating that it has low damage resistance. For CE mastic, the peak region is small, and there is a sudden reduction after the peak, starting at a 6% strain. ET and GE delay the falling of the curves at 8% strain and have the mildest falling slope compared to other mastics, with an extended plateau region at the peak zone. Mastic with LD filler shows a stress peak curve shape similar to that of the corresponding CE mastic (at 6.5% strain) but with lower stress levels. This could be due to the similar chemical composition.

**Figure 13:**
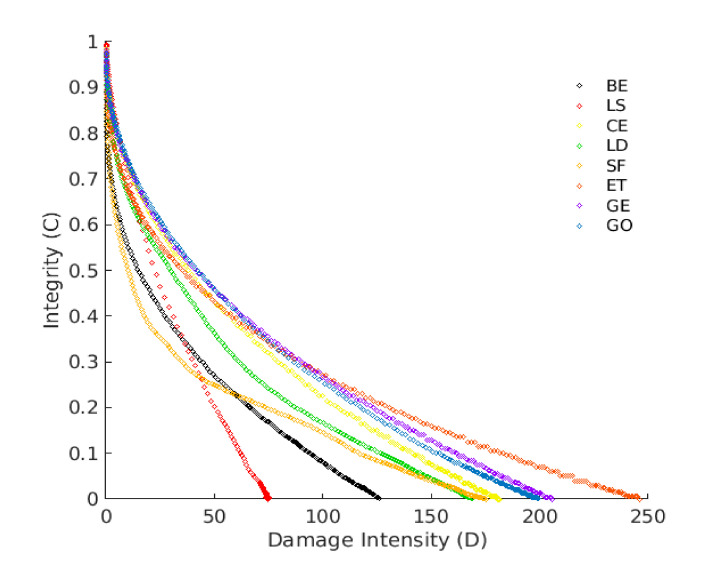
Viscoelastic Continuum Damage (VECD) curve from amplitude sweep.

However, LD contains lower CaO content (lower stiffening effect). Unlike the GE mastic, GO exhibited a very short plateau zone, and this difference could be attributed to the use of the chemical activator. Generally, active filler addition causes a phase inversion and forms a hydraulic binder network within the mastic matrix. This binder conveys more considerable elasticity to the mastic and, accordingly, greater resistance to rutting deformation, and could also offer good fatigue cracking resistance. For this reason, larger strain amplitudes are needed before the failure happens. These outcomes can be linked to filler’s chemical reactivity and physicochemical interaction, as discussed in Section 3.1 and Section 3.4. Figure 13 shows the damage characteristics (C-D) curves of the tested specimens. The C-D curve patterns are affected significantly by the filler inclusion, leading to mastics with different behavior. The parameter C refers to the integrity of the material, and it is assumed as 1.0 at the beginning of the test (materials without damage) and 0 (or nearly 0) at the end. The parameter D (damage intensity) indicates the quantity of work needed to decrease the C parameter.

Different mastics express different damage progression related to the resistance to fatigue damage. Based on the C-D curves, all mastics have improved damage resistance compared to the CBE binder except LS mastic as it exhibits a rapid degradation curve (accelerates the reduction of integrity parameter). This could be attributed to the LS filler’s low reactivity (inactive filler) within the CBE binder, as described in Figure 5. Meanwhile, the SF mastic shows early rapid damage at the initial stage. However, the rest of the mastics show a steadier reduction in parameter C. This implies that they have the ability to sustain far more damage than the BE, SF, and LS. Taking GE mastic as an example, it exhibits higher material integrity at a specific D value, indicating a better fatigue resistance. However, although ET mastic is stiffer than mastic GE, the loss in integrity is reached with a lower damage amount. CE mastic exhibits a lower capability to withstand additional deformation levels recognized by the earlier reduction in shear stress after approaching the peak level (see Figure 11).

Consequently, the greater strain levels in CE mastic are counterpoised by a rapid decrease in the integrity parameter, leading to smaller damage intensity at the same C value. The curve trend for the brittle and stiff mastic (e.g., CE) is characteristically recognized by a higher peak of stress with a steep post-peak slope, signifying stiff fatigue failure. However, the damage evolution analysis cannot provide the entire story regarding fatigue resistance. Therefore, the fatigue power-law model is fitted to integrity damage characteristics and utilized to obtain the fatigue laws and lives to compare the LAS results with TS approaches, as illustrated in Figure 9 and Table 2.

#### 3.3.3. Fatigue Results Comparison

Figure 9 and Table 2 show and compare fatigue laws and lives, respectively, obtained from TS and LAS tests. Generally, the predicted fatigue resistance with the time sweep test is longer than that of the LAS predictions, which agrees with other researchers [44]. The exception is BE and LS mastic, which have similar performance in the TS and LAS analyses. This is because the LAS test is based on an accelerated procedure; thus, the intensity of damage for the same C reduction is higher. Moreover, both tests are subjected to different effects (reversible phenomena), which are not taken into consideration in the study. As is known, the TS test is influenced by nonlinearity and thixotropy, while the LAS test is only influenced by nonlinearity. Therefore, they provide very different laws. However, both lines offer comparable outcomes about filler impact. From Figure 9, it can be observed that the fatigue life of CBE and all mastics decrease considerably as the strain increases. Despite slight differences between the fatigue laws for the same mastic, the ranking of mastics is the same for each approach (used to analyze TS data). Besides, it is observed that the filler type has a strong effect on fatigue law. Generally, each filler leads to a remarkable improvement of fatigue performance compared to the CBE binder, knowing that the used fillers increase the complex shear modulus of CBE significantly, as illustrated in Figure 10. Commonly, in the strain-controlled time sweep test, mastic with higher complex shear modulus (due to solid particle reinforcement) presents lower fatigue resistance. The reason is that larger particle to particle contact leads to greater complex shear modulus, which in turn causes a rise in the stress levels, and so the mastic withstands a smaller amount of damage at failure (fragile response).

Nonetheless, this effect is filler content dependent. In this regard, Liao et al. [45] found that the fatigue laws of mastics with diluted suspension have no dependency on the testing mode (controlled strain or stress), whereas the testing mode has an effect on the mastics with concentrated suspension. In this experimental work, low filler content was used to obtain dilute suspension mastics to eliminate the effect of the particle to particle contact phenomena. Furthermore, Little and Petersen [46] found that mastic modified with active fillers (i.e., hydrated lime) exhibited greater complex shear modulus and presented higher fatigue resistance in a controlled strain mode due to its higher ability to hold larger damage levels at failure than the neat binder. Thus, using active filler led to an increase in complex shear modulus and the elastic behavior, as stated in the viscoelastic analyses, and this was not accompanied by a significant decrease in the ductility for specific fillers.

### 3.4. Physicochemical Interaction of CBE Mastic

As mentioned previously, fillers reinforce CBE mastic through three main mechanisms: volume filling (filler density), particle geometry, and chemical interaction. The first two mechanisms are considered as mechanical reinforcement. The last mechanism includes filler affinity to bitumen (basic or acidic nature) and the way in which it interacts with CBE binder (adhesion between the particle and bitumen, and hydraulic binder network). Regarding the volume filling effect, low filler content was used in this work in order to obtain dilute suspension mastics to eliminate the effect of the particle to particle contact phenomena. Therefore, filler reactivity was expected to dominate the CBE mastic performance. From Table 1 and Table 3, it can be observed that low-density filler, such as SF filler, increase the complex shear modulus and decrease the phase angle as more solid particles are added to the matrix (no expected reactivity effect as SF is a low reactive filler when it is used alone in mastic) with moderate fatigue life. On the contrary, a filler such as CE (high reactivity and density), leads to very stiff materials even with low particle concentration due to the formed rigid hydration products. This consequence is clearly reflected in CE mastic’s fatigue resistance, as shown in Table 3. On the other hand, an inert filler such as LS, which also has relatively high density, exhibits lower complex shear modulus and a higher phase angle with poor fatigue life. Normally, in the case of inert filler, the complex shear modulus comes from solid particle inclusion (no or little chemical reaction, see Figure 5). Thus, the low fatigue life is due to the poor reactivity of LS filler (poor affinity of LS particles to bitumen, which weakens and divides the continuous phase of bitumen medium). However, this is not the case with active filler, as the hydraulic binder after the hydration process controls the mastic’s rheological behavior. Therefore, the expected improvement in fatigue resistance in this study mainly comes from the chemical interaction. In this concern, filler chemistry could play a crucial role in affecting the fatigue life of mastic.

ET filler offers an excellent fatigue life as it contains SO_3_ oxide (gypsum), which is usually incorporated for the purpose of regulating the setting time of hydration in cementitious binders to control the tricalcium aluminate (C3A) reaction. C3A is one of the main constituents of the cementitious binder that strongly reacts with water and leads to rapid setting. Therefore, SO_3_ sources, such as gypsum, are generally added to cement to control C3A hydration. In the presence of SO_3_, a calcium sulfoaluminate compound named ettringite is formed. The ettringite is a fine-grained crystal and forms a coating on the surface of the C3A particles. These crystals are not large enough to bridge the gaps between the cement particles, but provide the bitumen matrix with a reinforcement network [47,48]. Therefore, mastic with ET filler has the ability to hold more damage before failure. Besides, the physiochemical interaction also includes chemical compatibility between the active filler and the emulsifier molecules (surfactant head and tail) from the emulsion. Higher compatibility leads to the ultimate potential reaction between the emulsifier and the carbonate anion (from the active filler), forming salt at the interface zone. This salt can act as an adhesion enhancer (increase the particle-bitumen affinity) [49]. Thus, an appropriate choice of filler with suitable emulsifiers could act as a layer using the water from the aggregate surface, and help in the fast curing process.

## 4. Conclusions

In this study, the rheological characteristics and fatigue damage performance of CBE binder and mastics with different fillers were studied with the aid of a chemical reactivity test (rise in pH test). Furthermore, fatigue damage performance was analyzed by time sweep tests (five approaches) and by linear amplitude sweep test (VECD concept). In this framework, the following findings were drawn:

- The chemical analysis was effectively used to identify the filler alkalinity and affinity to bitumen emulsion. It was found that the pH level was dependent on specific oxides content (i.e., CaO and SiO_2_). This observation can be employed with filler physical properties to estimate the filler influence on mastic rheological and fatigue characteristics.
- Low filler reactivity (i.e., LS) maintained the viscoelastic behavior because of added inert solid particles to the system, but the fatigue life was reduced due to fragile behavior.
- The fatigue resistance of the CBE binder was enhanced significantly by incorporating active fillers. However, fillers with high reactivity, such as CE filler, exhibited stiffer behavior at low temperature (high frequencies), which illustrated why the CE mastic had low fatigue crack resistance.
- Ettringite based binder (calcium sulfoaluminate binder) provided a mastic matrix with a dense crystalline network, which increased the complex modulus and decreased the phase angle at low and moderate frequencies. The obtained reinforcement interrupted the crack initiation, causing a significant improvement in fatigue crack resistance.
- Both TS and LAS tests were successfully applied to the CBE mastic and gave comparable results. The expected fatigue lives with time sweep tests were generally longer than that of the LAS predictions. However, both testing procedures showed the same outcomes regarding filler impact (similar overall ranking).

Engineering of active fillers (with suitable emulsifier type) in CBE mastic could improve the early mechanical properties and also the fatigue resistance. Accordingly, the CBE mixture’s performance is correlated to more than one factor simultaneously occurring. Balancing these factors is the key to enhancing fatigue damage resistance.

## Figures and Tables

**Table 1:** Properties and chemical composition (wt%) of used fillers.

**Filler**	**SiO_2_**	**Al_2_O_3_**	**CaO**	**SO_3_**	**CaCO_3_**	**Fe_2_O_3_**	**Other Oxides (%)**	**Density (g/cm^3^)**	**^1^ ∆ R&B** **(°C)**
LS	0.53	0.16	0	0.08	98.03	0.08	1.12	2.73	1
CE	20.86	4.97	64.74	3.30	-	3.86	2.27	3.14	2.5
LD	8.33	28.91	52.41	1.83	-	1.39	7.13	2.58	1
SF	97.57	0.06	0.71	0.12	-	0.06	1.48	2.27	3
ET	6.29	20.30	49.18	18.02	-	1.02	5.18	2.62	2
GE	31.29	25.30	29.22	1.02	-	6.27	6.91	2.46	2
GO	31.29	25.30	29.22	1.02	-	6.27	6.91	2.46	2

^1^ Mastics were prepared with hot bitumen (same grade) with the same bitumen to filler ratio.

**Table 2:** The fatigue life of different mastics determined with different approaches.

Sample	Strain Level (%)	Fatigue Failure Criteria					
		RDEC	G_50%_	DER	DPA	ESR	LAS
	1.5	5930	6325	6151	6142	5805	6766
BE	2.5	1810	1540	1612	1636	1827	1875
	3.5	828	608	668	684	853	805
	1.5	1738	2219	1634	1369	2093	4843
LS	2.5	781	1052	746	742	970	1234
	3.5	462	643	445	496	585	501
	1.5	14,661	31,745	23,768	19,978	31,967	16,704
CE	2.5	7124	8767	8007	7011	9658	4368
	3.5	4428	3756	3910	3517	4391	1806
	1.5	17,347	21,148	19,847	22,298	24,098	11,636
LD	2.5	5239	5757	5346	5739	6517	3087
	3.5	2381	2444	2253	2347	2754	1288
	1.5	36,011	44,432	39,389	42,515	51,422	22,413
SF	2.5	10,434	11,244	10,778	10,721	12,900	2837
	3.5	4614	4548	4590	4326	5188	745
	1.5	143,888	109,384	131,017	98,243	147,797	30,629
ET	2.5	34,599	30,549	34,893	30,203	49,106	6544
	3.5	13,532	13,186	14,597	13,888	23,765	2368
	1.5	40,124	50,493	47,179	42,030	50,728	23,042
GE	2.5	15,811	19,287	18,497	16,767	31,739	5857
	3.5	8562	10,232	9983	9153	23,305	2377
	1.5	53,856	73,435	55,072	48,932	63,505	22,439
GO	2.5	16,719	18,736	16,673	14,331	17,502	5651
	3.5	7737	7620	7590	6382	7489	2278

**Table 3:** Complex shear modulus, phase angle, and Nf (G_50%_) of corresponding mastics at 10 Hz and 20 °C.

Sample	Complex Shear Modulus (Pa)	Phase Angle (°)	Nf (G50%)
BE	3.48 × 106	57	1.54 × 103
LS	2.78 × 106	55	1.05 × 103
CE	9.73 × 106	54	8.77 × 103
LD	3.37 × 106	59	1.87 × 104
SF	6.52 × 106	48	1.12 × 104
ET	1.04 × 107	49	3.05 × 104
GE	4.00 × 106	56	1.93 × 104
GO	6.00 × 106	55	5.76 × 103

## References

[1] Giani M.I., Dotelli G., Brandini N., Zampori L. (2015). Comparative life cycle assessment of asphalt pavements using reclaimed asphalt, warm mix technology and cold in-place recycling. Resour. Conserv. Recycl..

[2] Turk J., Mauko Pranjić A., Mladenovič A., Cotič Z., Jurjavčič P. (2016). Environmental comparison of two alternative road pavement rehabilitation techniques: Cold-in-place-recycling versus traditional reconstruction. J. Clean. Prod..

[3] Lesueur D., Teixeira A., Lázaro M.M., Andaluz D., Ruiz A. (2016). A simple test method in order to assess the effect of mineral fillers on bitumen ageing. Constr. Build. Mater..

[4] Yan K.Z., Bin Xu H., Zhang H.L. (2013). Effect of mineral filler on properties of warm asphalt mastic containing Sasobit. Constr. Build. Mater..

[5] Al-Mohammedawi A., Mollenhauer K. (2020). Viscoelastic Response of Bitumen Emulsion Mastic with Various Active Fillers. Proceedings of the 9th International Conference on Maintenance and Rehabilitation of Pavements and Technological Control, MAIERAPAV9.

[6] Buczyński P., Iwański M. (2017). Inactive Mineral Filler as a Stiffness Modulus Regulator in Foamed Bitumen-Modified Recycled Base Layers. IOP Conf. Ser. Mater. Sci. Eng..

[7] Kakade V.B., Reddy M.A., Reddy K.S. (2018). Rutting performance of hydrated lime modified bituminous mixes. Constr. Build. Mater..

[8] Temuujin J., van Riessen A., Williams R. (2009). Influence of calcium compounds on the mechanical properties of fly ash geopolymer pastes. J. Hazard. Mater..

[9] Dutta D., Thokchom S., Ghosh P., Ghosh S. (2010). Effect of silica fume additions on porosity of fly ash geopolymers. J. Eng. Appl. Sci..

[10] Steveson M., Sagoe-Crentsil K. (2005). Relationships between composition, structure and strength of inorganic polymers. J. Mater. Sci..

[11] Criado M., Aperador W., Sobrados I. (2016). Microstructural and mechanical properties of alkali activated Colombian raw materials. Materials.

[12] Choi S.C., Lee W.K. (2012). Effect of Fe_2_O_3_ on the physical property of geopolymer paste. Adv. Mater. Res..

[13] Clopotel C., Velasquez R., Bahia H. (2012). Measuring physico-chemical interaction in mastics using glass transition. Road Mater. Pavement Des..

[14] Kim M., Buttlar W.G. (2010). Stiffening mechanisms of asphalt-aggregate mixtures: From binder to mixture. Transp. Res. Rec..

[15] Underwood B.S., Kim Y.R. (2011). Experimental investigation into the multiscale behaviour of asphalt concrete. Int. J. Pavement Eng..

[16] Rigden P.J. (1947). The use of fillers in bituminous road surfacings. A study of filler-binder systems in relation to filler characteristics. J. Soc. Chem. Ind..

[17] Nikolaides A.F. (2015). Bituminous Mixtures & Pavements VI. Proceedings of the 6th International Conference on Bituminous Mixtures and Pavements, ICONFBMP 2015.

[18] Fang X., Garcia A., Lura P. (2016). Overview on cold cement bitumen emulsion asphalt. RILEM Tech. Lett..

[19] Dai Q., Sadd M.H. (2004). Parametric Model Study of Microstructure Effects on Damage Behavior of Asphalt Samples. Int. J. Pavement Eng..

[20] Anderson D.A., Goetz W.H. (1973). Mechanical behavior and reinforcement of mineral filler-asphalt mixtures. J. Assoc. Asph. Paving Technol..

[21] Anderson D.A., Tarris J.P., Brock J.D. (1982). Dust collector fines and their influence on mixture design. J. Assoc. Asph. Paving Technol..

[22] Kandhal P.S. (1981). Evaluation of baghouse fines in bituminous paving mixtures (with discussion). Assoc. Asph. Paving Technol. Proc..

[23] Ziyani L., Gaudefroy V., Ferber V., Deneele D., Hammoum F. (2013). Chemical reactivity of mineral aggregates in aqueous solution: Relationship with bitumen emulsion breaking. J. Mater. Sci..

[24] Zizi Z., Oulahna D., Benhassaine A., Sainton A., Pelon M. (1997). Emulsion—Fine siliceous solids’ system. The physical breaking of the emulsion. Bull. Lab. Ponts Chaussees.

[25] Gaestel C. (1967). The breaking mechanism of cationic bitumen emulsions. Chem. Ind..

[26] Luo R., Zhang D., Zeng Z., Lytton R.L. (2015). Effect of surface tension on the measurement of surface energy components of asphalt binders using the Wilhelmy Plate Method. Constr. Build. Mater..

[27] Al-Mohammedawi A., Mollenhauer K., Di Benedetto S., Baaj H., Chailleux H., Tebaldi E., Sauzeat G., Mangiafico C. (2020). A Study on The Fatigue Behavior Of Bitumen Emulsion Mastic, Modified with Various Active Fillers. Proceedings of the RILEM International Symposium on Bituminous Materials.

[28] Mazzoni G., Stimilli A., Cardone F., Canestrari F. (2017). Fatigue, self-healing and thixotropy of bituminous mastics including aged modified bitumens and different filler contents. Constr. Build. Mater..

[29] Marasteanu M., Ghosh D., Falchetto A.C., Turos M. (2017). Testing protocol to obtain failure properties of asphalt binders at low temperature using creep compliance and stress-controlled strength test. Road Mater. Pavement Des..

[30] Tayebali A.A., Rowe G.M., Sousa J.B. (1992). Fatigue response of asphalt-aggregate mixtures. J. Assoc. Asph. Paving Technol..

[31] Pronk A.C. Comparison of 2 and 4 point fatigue tests and healing in 4 point dynamic bending test based on the dissipated energy concept. Proceedings of the 8th International Conference on Asphalt Pavements.

[32] Rowe G.M. (1996). Application of the Dissipated Energy Concept to Fatigue Cracking in Asphalt Pavements. Ph.D. Thesis.

[33] Mitchell D., Witczak M., Mamlouk M., Kaloush M., Kaloush K. (2007). Validation of Initial and Failure Stiffness Definitions in Flexure Fatigue Test for Hot Mix Asphalt. J. Test. Eval..

[34] Kim Y.R., Little D.N., Lytton R. (2003). Fatigue and Healing Characterization of Asphalt Mixtures. J. Mater. Civ. Eng..

[35] Wen H., Bahia H. (2009). Characterizing Fatigue of Asphalt Binders with Viscoelastic Continuum Damage Mechanics. Transp. Res. Rec..

[36] Nguyen H., Adesanya E., Ohenoja K., Kriskova L., Pontikes Y., Kinnunen P., Illikainen M. (2019). Byproduct-based ettringite binder—A synergy between ladle slag and gypsum. Constr. Build. Mater..

[37] Kim Y.R. (2009). Modeling of Asphalt Concrete.

[38] Papagiannakis A.T., Masad E.A. (2008). Pavement Design and Materials.

[39] Rowe G.M., Bouldin M.G. Improved Techniques to Evaluate the Fatigue Resistance of Asphaltic Mixtures. Proceedings of the 2nd Eurasphalt Eurobitume Congress.

[40] Hintz C., Velasquez R., Johnson C., Bahia H. (2011). Modification and validation of linear amplitude sweep test for binder fatigue specification. Transp. Res. Rec..

[41] Johnson C. (2010). Estimating Asphalt Binder Fatigue Resistance Using an Accelerated Test Method. Ph.D. Thesis.

[42] Johnson C., Bahia H. (2010). Evaluation of an accelerated procedure for fatigue characterization of asphalt binders. Road Mater. Pavement Des..

[43] Schapery R.A. (1984). Correspondence principles and a generalized J integral for large deformation and fracture analysis of viscoelastic media. Int. J. Fract..

[44] Bessa I., Vasconcelos K., Branco V.T.F.C., Bernucci L.L.B. (2019). Fatigue resistance of asphalt binders and the correlation with asphalt mixture behaviour. Road Mater. Pavement Des..

[45] Liao M.C., Chen J.S., Tsou K.W. (2012). Fatigue characteristics of bitumen-filler mastics and asphalt mixtures. J. Mater. Civ. Eng..

[46] Little D.N., Petersen J.C. (2005). Unique Effects of Hydrated Lime Filler on the Performance-Related Properties of Asphalt Cements: Physical and Chemical Interactions Revisited. J. Mater. Civ. Eng..

[47] Mehta P.K. (1969). Morphology of Calcium Sulfoaluminate Hydrates. J. Am. Ceram. Soc..

[48] Quennoz A. (2011). Hydration of C_3_A with Calcium Sulfate Alone and in the Presence of Calcium Silicate. EPFL.

[49] Sjoblom J. (2005). Emulsions and Emulsion Stability: Surfactant Science Series/61.

